# Highly Conserved Non-Coding Sequences and the 18q Critical Region for Short Stature: A Common Mechanism of Disease?

**DOI:** 10.1371/journal.pone.0001460

**Published:** 2008-01-23

**Authors:** Flavio Rizzolio, Silvia Bione, Cinzia Sala, Carla Tribioli, Roberto Ciccone, Orsetta Zuffardi, Natascia di Iorgi, Mohamad Maghnie, Daniela Toniolo

**Affiliations:** 1 Department of Biotechnologial Research (DIBIT), San Raffaele Scientific Institute, Milano, Italy; 2 Institute of Molecular Genetics, Consiglio Nazionale delle Ricerche (CNR), Pavia, Italy; 3 Medical Genetics, University of Pavia, Pavia, Italy; 4 Department of Pediatrics, Istituti di Ricovero e Cura a Carattere Scientifico (IRCCS) G. Gaslini, University of Genova, Genova, Italy; University of Minnesota, United States of America

## Abstract

**Background:**

Isolated growth hormone deficiency (IGHD) and multiple pituitary hormone deficiency (MPHD) are heterogeneous disorders with several different etiologies and they are responsible for most cases of short stature. Mutations in different genes have been identified but still many patients did not present mutations in any of the known genes. Chromosomal rearrangements may also be involved in short stature and, among others, deletions of 18q23 defined a critical region for the disorder. No gene was yet identified.

**Methodology/Principal Findings:**

We now report a balanced translocation X;18 in a patient presenting a breakpoint in 18q23 that was surprisingly mapped about 500 Kb distal from the short stature critical region. It separated from the flanking *SALL3* gene a region enriched in highly conserved non-coding elements (HCNE) that appeared to be regulatory sequences, active as enhancers or silencers during embryonic development.

**Conclusion:**

We propose that, during pituitary development, the 18q rearrangement may alter expression of 18q genes or of X chromosome genes that are translocated next to the HCNEs. Alteration of expression of developmentally regulated genes by translocation of HCNEs may represent a common mechanism for disorders associated to isolated chromosomal rearrangements.

## Introduction

IGHD and MPHD are endocrine disorders responsible of most cases of short stature. They may have different etiologies and are often associated with structural hypothalamic-pituitary (H-P) defects detectable by neuroimaging [1], [2]. Both IGHD and MPHD may have a genetic origin, as shown by identification of mutations in genes encoding critical components of the H-P axis. Two genes, the *GH1* encoding the GH and the *GHRHR*, expressed along the somatotropic axis and six genes encoding transcription factors involved in anterior pituitary gland development (*POU1F1, PROP1, HESX1, LHX3, LHX4* and *SOX3*), have been identified in patients with GH deficiency [1], [2]. However, it is likely that other genes may be involved in the etiology of GH-dependent short stature, as the majority of patients did not present mutations in any of the known genes [3], [4].

As it was the case for many other heterogeneous disorders, chromosomal variations may also be involved in determining the short stature phenotype. It is well established that relatively common chromosomal rearrangements associated with short stature are 18q deletions [5]. The cytogenetic and molecular localization of the deletions in a large number of patients demonstrated a common deleted region of about 2 Mb, defined as the critical region for short stature [6]. The same region was recently confirmed and precisely defined by array CGH analysis. In the same study, two additional commonly deleted regions, localized more proximally along 18q, were identified [7]. In the few cases when it was tested, the GH deficiency resulted to depend from a defect in hypothalamic or neurosecretory functions that control pituitary GH synthesis [5], [8]. In only one case a pituitary malformation was reported [9]. However, the deletion of the critical region was not always sufficient to cause short stature as a number of patients presented with stature in the normal range. Moreover, 18q partial monosomy resulted in variable severity of the phenotype that did not correlate with the size of the deletions. Altogether the data may indicate that haploinsufficiency for one gene in 18q23 may cause GH deficiency and short stature, but that it likely represents a risk factor rather than a cause for the disorder.

Among the four genes in the critical region (two ZNF proteins encoding genes, *ZNF516* and *ZNF236,* the *MBP* and *GALR1* genes) *GALR1* was considered the best candidate. *GALR1* is one of the receptors of Galanin, a 29 aminoacid neuropeptide playing a critical role in many diverse central and peripheral nerve functions [10]. The biological effects of galanin are mediated by three G protein coupled receptors, GalR1, GalR2 and GalR3, that show a widespread distribution throughout the central and peripheral nervous system and may participate in different aspects of the galanin function [11]. Their specific role has not yet been determined. From the study of a *Galr1* KO mice [12] it seems that it is mainly involved in the neuroprotective action of galanin and in its anticonvulsant effect, while it does not seem to be involved in other galanin functions such as nociception. The KO mice did not appear to have any hypothalamic or pituitary related defects [12]. In conclusion the role of *GALR1* in GH deficiency is far from being clarified but from the phenotype of the KO mice it is not unlikely that other genes in the region should be considered as candidates for short stature.

One female patient presenting with GHD and ectopic posterior pituitary at Magnetic Resonance Imaging (MRI) was described. She carried an X;18 balanced translocation with breakpoints in Xq22.3 and 18q23 [13]. The patient had primary amenorrhea, that had been ascribed to interruption of the Xq Premature Ovarian Failure (POF) critical region, while the short stature was related to the 18q rearrangement.

Here we present the fine mapping of both Xq and 18q breakpoints in the patient and we report that in 18q it was localized outside and distally from the short stature critical region. It was mapped 80 Kb upstream from a gene, *SALL3*, a developmentally regulated transcription factor that seems to act as downstream targets of hedgehog. It had been previously considered as a candidate for different phenotypes associated to the 18q- syndrome, but not for short stature, as it was not deleted in one patient [5], [7]. The analysis of the 18q breakpoint highlighted a gene free region enriched in HCNEs and indicated that translocation of the HCNEs itself may be responsible for the patient phenotype.

## Results

### Fine mapping of the X/18 breakpoints

**Figure 1:**
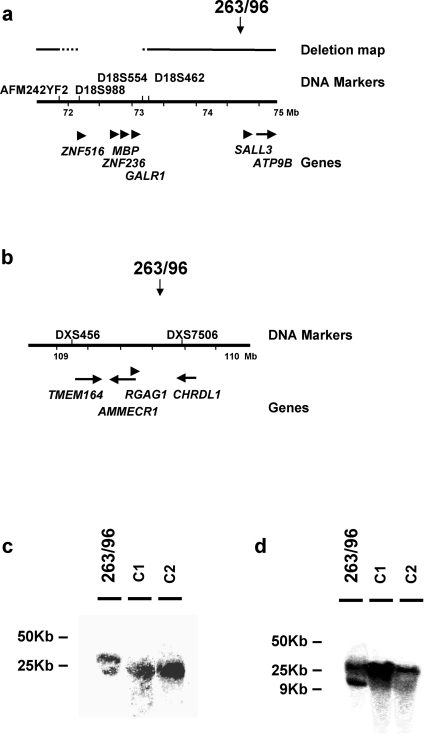
Breakpoint map of the 263/96 patient. a. Map of the 18q breakpoint region. The position of the breakpoint at 74.8 Mb of chromosome 18 is indicated by a vertical arrow. Below is a schematic representation of the deletion map and of the short stature critical region [6], [7]. The position of the markers used is indicated; the critical region boundaries are indicated by dotted lines. Under the map are the genes in the region: horizontal arrows represent transcription orientation. b. Map of the Xq breakpoint region. The position of the breakpoint at 109.7 Mb of chromosome X is indicated by a vertical arrow. Under the map are the genes in the region: horizontal arrows represent transcription orientation. c. Southern Blot analysis of the 18q breakpoint: DNA from the patient and of two normal DNAs was digested with *BamH1* and fractionated by PFGE as described previously [31]. The Blot was hybridized with the probe 263/5. d. Southern Blot analysis of the Xq breakpoint: DNA from the patient and of two normal DNAs was digested with *KpnI* and fractionated by PFGE as described previously [31]. The Blot was hybridized with the probe 263×1. Probes are described in Materials and Methods. Map positions are from NCBI Release 36.1

The 18q breakpoint in the patient 263/96 was mapped by FISH to a 140 Kb region defined by BAC RP11-496G14, that hybridized to chromosome 18 and to der18 and by BAC RP11-850021 that hybridized to chromosome 18 and to derX (not shown): genome sequence analysis mapped the breakpoint distal from the *GALR1* gene and proximal to the *SALL3* gene (Fig. 1a). The X chromosome breakpoint was similarly mapped by FISH (Fig. 1). The PAC RP1-302C5, in Xq23, was found to hybridize to chromosome X and to the two derivative chromosomes [14].

Both breakpoints were defined by PFGE analysis (Fig. 1 c and d). Genomic DNA of the patient and of controls were digested with the *Bam*HI and *Kpn*I restriction enzymes to map the 18q and Xq breakpoints respectively. Genomic probes in the regions hybridized to *Bam*HI and *Kpn*I fragments of the expected size. The genomic probe 263/5, from chromosome 18, hybridized to a 15 Kb fragment in all samples, and to an additional fragment of 26 Kb in the patient DNA (Fig. 1c) indicating that the breakpoint mapped less than 15 Kb from the *Bam*HI site and 80 Kb from the 5′ end of the *SALL3* gene. The genomic probe 263×1 (chromosome X) hybridized to a 23 Kb fragment in all samples, and to an additional fragment of 10 Kb in the patient DNA (Fig. 1d) indicating that the breakpoint mapped 40 Kb downstream from the *RGAG1* gene and 180 Kb from the 3′ end of the *CHRDL1* genes (Fig. 1b).

A genome-wide array-CGH analysis did not show any additional genomic imbalance, with the exception of known copy number variations already reported on the Database of Genomic Variants (http://projects.tcag.ca/variation/). Considering the average spacing among probes and that at least three consecutive probes with a shifted log_2_ ratio are needed to make a call for an imbalance, we could not identify any deletion larger than 20 Kb, on average.

In conclusion, none of the breakpoints interrupted a gene and no other rearrangement was detected in the patient. Moreover the 18q breakpoint mapped outside from the critical region defined from deletion mapping (Fig. 1a). The distal boundary of the region was defined by few patients carrying interstitial deletions and particularly by patient n°13 from Cody et al. [6], presenting a distal breakpoint between 73.1 and 73.4 Mb, >1 Mb from that in the patient.

### Comparative genome analysis of the breakpoint regions

**Figure 2:**
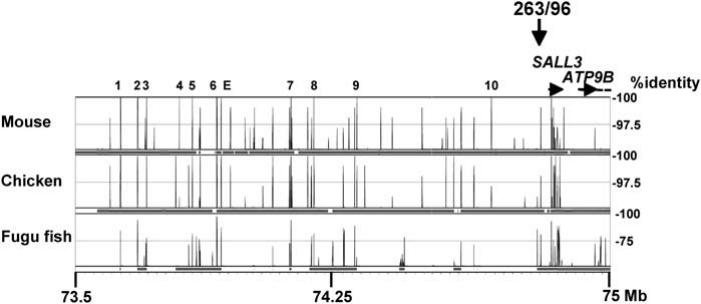
Comparative analysis of the HCNE rich region in 18q. The portion of the analysis from the VISTA Genome Browser (http://genome.lbl.gov/vista/index.shtml) from 73,5 to 75 Mb of human chromosome 18 is reported to show conservation between human and mouse, chicken or fugu. The HCNEs are numbered above the peaks. The position of the 263/96 breakpoint is indicated by a vertical arrow, that of the genes *SALL3* and *ATP9B* by a horizontal arrow. The homology between human and the species indicated is shown on the right (%). Map positions in human correspond to NCBI Release 35.

To find an explanation for the phenotype of the patient, we looked for evolutionary conserved non-coding sequences that may have a regulatory function on gene expression. We compared the DNA sequences flanking the patient breakpoints to the syntenic regions of mouse, chicken and fugu utilizing the VISTA Genome Browser (http://genome.lbl.gov/vista/index.shtml). No highly conserved sequences were found at the Xq breakpoint region. On the other hand, in the 2 Mb gene free region between *GALR1* and *SALL3* several highly conserved non-coding elements (HCNE), could be detected [15], [16], [17]. Among the HCNEs, 11 presented 100% identity over 100 bp between human and mouse and at least 97% identity over 100 bp between human and chicken (Fig. 2). They were clustered in a 700 Kb region, 500 Kb centromeric to the *SALL3* gene. All were localized about 500 Kb distal from the short stature critical region and distal in respect to an “evolutionary breakpoint” at 73.5 Mb of human chromosome 18, where synteny between chicken and mammals ended (Fig. 2). None of the HCNEs could be defined ultraconserved (UCR), e.g. presenting 100% identity between human and mouse for at least 200 bp [15], [16]. However some were highly conserved in Fugu: HCNE 2, 6, and 7 had >85% identity for >100 bp between fugu and human (Fig. 2).

### Functional analysis of the HCNEs

**Figure 3:**
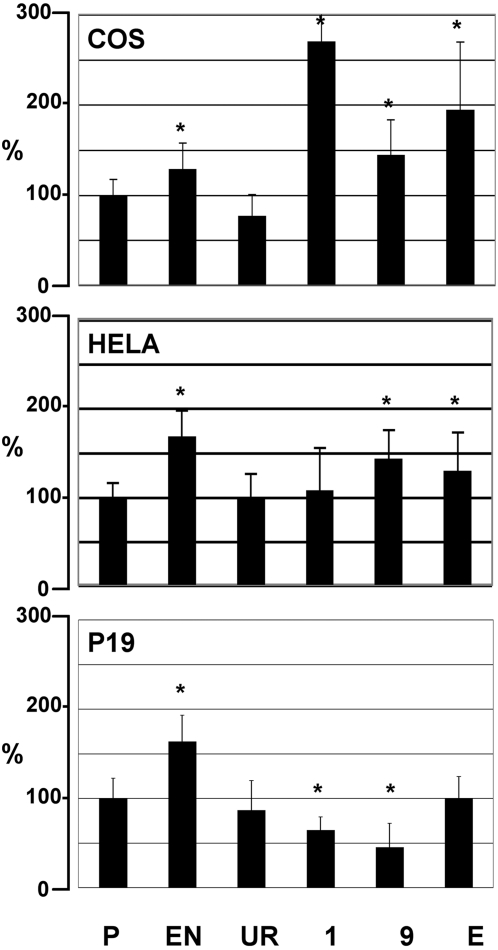
Some of the HCNEs have enhancer or silencer function. Luciferase report assays of the cell lines indicated, transfected with constructs containing promoter only vector (P), a conserved elements from a different genomic region, showing enhancer activity (EN), an unrelated negative control (UR: human/mouse non-conserved element), HCNE1, 9 and E as in Fig. 2. All values are expressed as % of the luciferase activity of the promoter vector (P). *: p value<0.001.

HCNEs were previously shown to have enhancer function [18]. Seven of the HCNEs in the region (HCNE1, 3, 6, 7, 8, 9 and E, in Fig. 2) were cloned into the pGL2 luciferase vector [19], upstream of the SV40 promoter and tested in different cell lines. As shown in Fig. 3, three of the HCNEs had indeed enhancer activity and increased the luciferase activity from 1.5 to 3 times compared to the promoter only construct (P) in Hela and COS cells. HCNE 1, 9 and E demonstrated enhancer activity in COS cells: HCNE 1 and E more than doubled the luciferase activity. HCNE 9 had a lower enhancer activity (1.5 times the promoter only construct) that was maintained in Hela cells. In a third cell line, P19, of neural origin, none of the HCNEs had enhancer activity, but HCNE 1 and HCNE 9 significantly decreased the luciferase activity, behaving as gene silencers.

From these in vitro experiments we can conclude that the HCNEs upstream from the *SALL3* gene may be regulatory elements controlling the expression of genes in the region either as enhancer or silencers.

### Chromatin modifications of HCNEs during mouse development

HCNEs were often found in the vicinity of developmentally expressed genes and it was shown that they may be developmental specific enhancers [18]. It was also shown that in embryonic stem cells HCNE rich loci presented a characteristic chromatin modification pattern, termed “bivalent domain” consisting of large regions of tri-methyl-lysin 27 histone 3 (3MK27H3) harboring smaller region of di-methyl-lysin 4 histone 3 (2MK4H3), suggestive of a poised state for activation [17].

**Figure 4:**
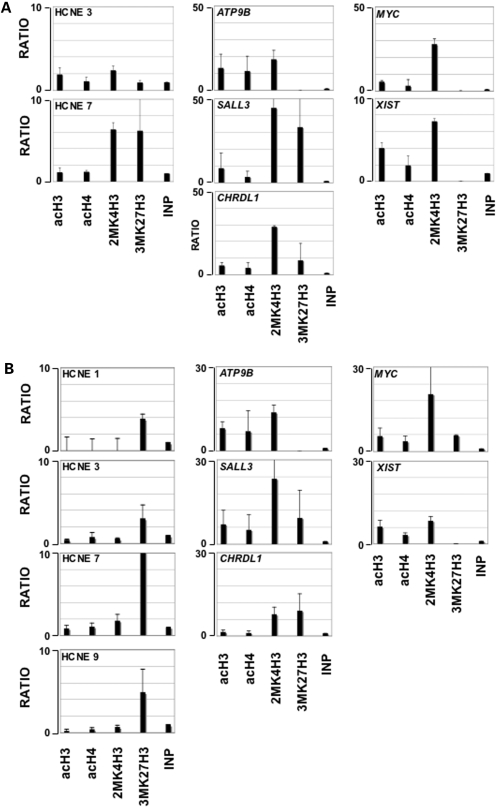
ChIP of the HCNE on total E11.5 chromatin and adult mouse brain. Embryonal (a) and adult brain chromatin preparations (b) were immunoprecipitated with the antibodies to the histone modifications indicated on the X axis and the DNA was PCR amplified with primers specific for each HCNE. INP: PCR from total chromatin. AcH3 and acH4: ChIP with antibodies to acetylated histone H3 or H4. 2MK4H3: ChIP with antibodies to 2-methyl K4 of histone H3; 3MK27H3: ChIP with antibodies to 3-methyl K27 of histone H3. All values are fold increases, compared to an equal amount of INP. *Myc*, *Xist* (positive controls), *Atp9b*, *Sall3* and *Chrdl1* indicate primers in the 5′UTR region of each gene. Only HCNEs positive for at least one modification were reported in the figure.

We analyzed by chromatin immunoprecipitation (ChIP) the acetylation of histone H3 (acH3) and H4 (acH4), and the methylation of K4 and K27 of histone H3 (2MK4H3 and 3MK27H3) of all the HCNEs in mouse embryo at E11.5 (Fig. 4a) to look for developmentally regulated histone modifications. Two controls, the promoters of the expressed genes *Myc* and *Xist*, were modified as expected by the active chromatin modifications, acH3, acH4 and 2MK4H3. Two HCNEs, HCNE3 and HCNE7, were found enriched in some of the histone modifications analyzed. HCNE3 was enriched in acH3 and 2MK4H3: it appeared to have an open chromatin conformation and it may be therefore an active enhancer at E11.5. HCNE7 was enriched for both 2MK4H3 and 3MK27H3 and may thus present the bivalent domain previously described for developmentally regulated HCNE. The same chromatin organization was found also at the promoter regions of the *Sall3* and *Chrdl1* genes (on mouse chromosomes 18 and X respectively) both flanking the breakpoints in the 263/96 patient and expressed at early developmental stages in the mouse [20], [21], [22]. A third gene analyzed, *Atp9b*, localized distally from *Sall3* but ubiquitously expressed, presented only open chromatin modifications.

For comparison we studied adult mouse brain (Fig. 4B). All the HCNEs were negative for open chromatin modifications and therefore they may not be active. Accordingly, many of the HCNEs (1, 3, 7, 9) showed high level of 3MK27H3, suggestive of a closed chromatin modification. All promoter regions had in adult brain the same modifications shown for E11.5.

In conclusion, as summarized in Table 1, the HCNEs between *Galr1* and *Sall3* appeared to be highly regulated elements, presenting an open chromatin conformation only during early stages of development. They seem to acquire a closed conformation in the adult, where they are presumably inactive.

**Table 1:** Summary of HCNE characteristics

HCNE	Enhancer°	Silencer°°	Chromatin modification*	
			E11.5	Adult brain
HCNE1	yes	yes	none detectable	inactive
HCNE3	no	no	active	inactive
HCNE7	no	no	active and inactive	inactive
HCNE9	yes	yes	none detectable	inactive
HCNEE	yes	no	none detectable	none detectable

° in COS and Hela cells

°° in P19 cells

* active: AcH3 and 2mK4H3; inactive: 3mK27H3

### *Sall3*, *Chrdl1* and *Atp9b* expression during mouse embryo development

**Figure 5:**
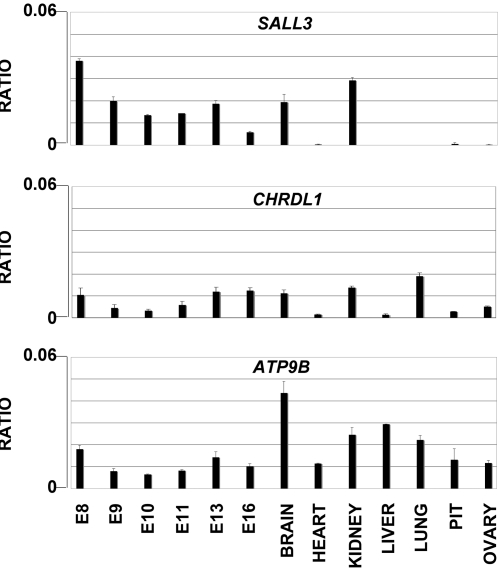
Real time RT-PCR of the *Chrdl1*, *Sall3* and *Atp9b* genes in mouse tissues. RNAs from the tissues and the developmental stages indicated on the X axis were reverse transcribed and PCR amplified as described in Materials and Methods. Each value was normalized as described in Materials and Methods and the ratio with the histone H3 gene expression was calculated.

The chromatin results suggested that the chromosomal rearrangement in the patient with GH deficiency might alter the expression of developmentally expressed genes flanking the breakpoints. The *Sall3* gene on chromosome 18 and the *Chrdl1* gene on the X chromosome have been reported previously to have a developmentally regulated expression [20], [23]. Analysis by real time RT PCR (Fig. 5) confirmed that *Sall3* is expressed at high level in mouse embryo until E15.5. At later stages it is down regulated and in the adult is expressed only in few tissues, brain and kidney among the one tested. It is not expressed in the adult pituitary. Also the *Chrdl1* on the X chromosomes is expressed at early developmental stages (E8) and only in some tissues in the adult. The third gene, *Atp9b*, distal from *Sall3* on mouse chromosome 18 was rather ubiquitously expressed in embryo and in all adult tissue analyzed (Fig. 5). Both *Atp9b* and *Chrdl1* are expressed at very low level in the ovary.

We analyzed expression of the three genes by *in situ* hybridization in the ovary and in the developing pituitary. *In situ* hybridization failed to show any specific hybridization in the ovarian follicle in adult (P20) mice and in E16.5 (not shown) that could account for the POF phenotype of the patients. *In situ* hybridization failed to show expression of the *Sall3* gene in at all stages of the developing pituitary, E9.5, E10.5, E12.5, E14.5 and E17.5 (not shown). The *Chrdl1* was faintly expressed in the pituitary at E17.5. *Atp9b* was expressed at low level at E14.5 and E17.5 (not shown).

In conclusion, as summarized in Table 2, the three genes appeared expressed during development and their regulated expression may be controlled or altered by the presence of flanking HCNEs.

**Table 2:** Summary of gene characteristics at E11.5

Genes	Chromatin modification*	Gene expression	
		whole embryo ˆ	pituitary°
*Sall3*	active and inactive	yes	no
*Atp9b*	active	yes(low)	no
*Chrdl1*	active and inactive	yes(low)	no

* active: AcH3 and 2mK4H3; inactive: 3mK27H3

ˆ By real time RT-PCR; comparison were with histone H3 mRNA

° By in situ hybridization

### The chromatin of the *SALL3* and *ATP9B* genes was altered in the lymphoblastoid cell line of the patients

**Figure 6:**
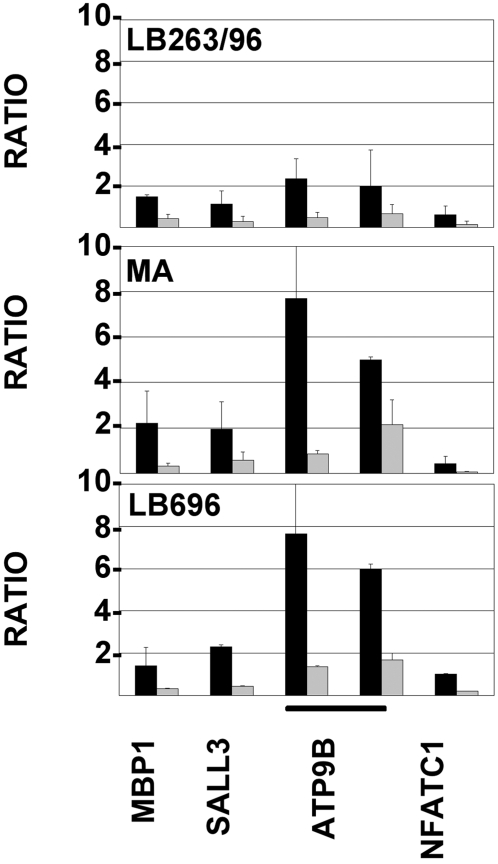
Chromatin modifications of genes flanking the 18q breakpoint, in the LB263/96 patient. Chromatin of the patient 263/96 and two normal lymphoblastoid cell lines was IP with antibodies to acH3, acH4 or to 2MK4H3 and the DNA was amplified by real time PCR with primers in the promoter regions of each gene. One or two sets of primers were utilized for each gene, as indicated. Modifications were calculated as described in Materials and Methods. In the figure, the ratio between acH3/acH4 (black) and acH4/2MK4 (gray) in chromatin from the LB263/96 patient and two normal controls (MA and LB696) is shown.

Chromatin prepared from the patient 263/96 and two normal lymphoblastoid cell controls was analyzed to establish whether the rearrangement might alter the organization of the promoters of the genes in the vicinity of the breakpoints. Antibodies to acH3, acH4 and 2MK4H3, markers of active chromatin state, were used for ChIP. The analysis was done by real time PCR with primers in the promoters of the genes flanking the breakpoints. Since the average chromatin modification level was not identical in different cell types, to compare results between different cell lines, we calculated the ratio of acH4/acH3 and acH4/2MK4H3 in each cell line (Fig. 6). We could not find chromatin modification in the promoter regions of the X chromosome genes *RGAG1* and *CHRDL1* nor for the *GALR1* gene. We were able to analyze *SALL3* and *ATP9B* genes and the genes more distant on chromosome 18 (*MBP* at the proximal side and *NFATC1* at the distal side). Translocation to the X chromosome critical region of *SALL3* and *ATP9B* caused a reduction of the acH4 modification compared to the controls (Fig. 6). AcH3 and 2MK4H3 were unchanged.

## Discussion

The results report the molecular analysis of the critical region for short stature in 18q and highlight a novel mechanism of disease that may be rather common when genomic regions presenting high evolutionary conservation [15] are involved in chromosomal rearrangements.

In the analysis of a X;18 balanced translocation in a patient affected with GHD and ectopic posterior pituitary, we were surprised to find that the 18q breakpoint did not interrupt the short stature critical region in 18q23 [6], [7]. Rather, it mapped about 1 Mb distally and 80 kb upstream from the *SALL3* gene promoter, at a first glance, pointing to *SALL3*, a gene outside of the critical region, as the candidate gene for the phenotype. To reconcile the contradictory data coming from the molecular definition of the chromosomal rearrangements we report here the observation of 11 HCNEs, presenting 100% conservation for at least 100 nt among mammals and >97% with chicken. All were clustered within 700 Kb where many highly conserved elements were present >90% identical within mammals. The HCNE cluster was localized in a gene desert between the promoter of *SALL3* and the *GALR1* gene: its proximal end, at about 73.6 Mb of chromosome 18, corresponded to the point where the synteny with chicken ended (Fig. 2). The data indicated that the HCNEs may be evolutionary conserved controlling elements of the *SALL3* gene, as it was suggested also by the finding of HCNEs in a similar position in two other members of the *SALL* gene family, *SALL1* and *SALL4* [18], [24].

HCNEs were often found in the vicinity of developmentally regulated genes and have been shown to function as developmental specific enhancers in several systems [18]. It was suggested that they may work by binding transcription factors and/or as organizers of genomic architecture around developmental genes. Indeed, the genes distal from the HCNE cluster, *SALL3* and *ATP9B*, were expressed early during development in the mouse starting from E8, the earliest stage studied. While the function of *ATPB* is not known, *SALL3,* a member of a family of genes with homology to the *spalt* gene of *Drosophila melanogaster*, was particularly interesting. *Spalt* genes encode transcription factors that in *Drosophila* act as downstream targets of hedgehog [25]. *Sal*-like genes were identified in vertebrates and they are all expressed during embryonic development: it was suggested that they may dictate the nuclear localization for correct gene transcription [24]. Finally, mutations in two members of the family (*SALL1* and *SALL4*) are responsible of human developmental disorders [25].

Our data is consistent with the idea that the HCNEs in 18q23 may function as enhancers (as in Hela or COS cells) as well as silencers (as in P19 cells), depending from the cell type considered. Analysis of chromatin modifications [26] demonstrated that, in mouse embryo chromatin at E11.5, some of the HCNEs presented active chromatin modifications that were absent in adult animals. One, HCNE3, had an active only chromatin state, and may thus carry an open chromatin conformation in most cells at the E11.5. The other, HCNE7, showed a bivalent domain of modifications with enrichment for both 2MK4H3 and 3MK27H3. This may results from chromatin in a poised state for transcription as suggested previously [17] or from different chromatin organization in the different cells of the E11.5 embryo. In any case, we can conclude that the HCNEs in the region are developmentally regulated enhancers/silencers, that may up or down regulate expression of flanking genes in a cell and stage specific way, as shown in vivo for similar genomic regions [18]

Among the genes in the region, *SALL3* was highly expressed in brain and it was previously suggested that it may be involved in determining some of the neurological phenotypes of the 18q deletion syndrome such as mental retardation, hearing loss, ophthalmic abnormalities [7]. In agreement with the finding that haploinsufficiency for *SALL3* does not seem to be involved with short stature, we show here that *Sall3* did not appear expressed in the developing nor in adult pituitary. We therefore propose that the balanced translocation will cause ectopic expression of *SALL3,* and possibly of distally flanking genes, in the pituitary through elimination of a silencing effect of the HCNEs. A similar mutation was demonstrated for the *Shh* gene whose ectopic expression in the anterior limb of the mouse was due to insertions about 1Mb upstream from *Shh*. The region contains a conserved non-coding element that can function as enhancer as well as a repressor and drives Shh expression in the limb. Its rearrangement was responsible for the phenotype of mouse mutants presenting with limb defects [27]. The 18q23 HCNEs may be the first example of such effect in a human disorder.

The phenotype of the patient could be due also to a more complex effect of the HCNEs, as a second interesting gene, was involved in the rearrangement, *CHRDL1*. The *CHRDL1* gene on the X chromosome encodes for an inhibitor of BMP signaling through its cysteine-rich repeats. Its expression pattern coincides with that of some members of the BMP signaling pathways [28], a large subgroup of the transforming growth factor beta superfamily, that serve key roles in stem cell fate commitment. Interestingly, some of the members of the BMP family (BMP4 and BMP2) appear to be involved in the Rathke's pouch formation [29] and pituitary development. In the mouse, the *Chrdl1* gene was expressed at low level in the pituitary at late stages of development and in the adult. Once translocated downstream to 18q23, this low level of expression in the pituitary could be either inhibited or enhanced by the upstream HCNEs. Deregulation of expression of *CHRDL1* and *SALL3* could therefore be factors in determining the phenotype of the patient. Moreover, it could explain the pituitary ectopia which is not a common feature of the GH deficiency and short stature, as it was described only once in the 18q- syndrome [9].

The X;18 patient had hypergonadotropic primary amenorrhea and ovarian dysgenesis [13]. Due to the involvement of X chromosome POF critical region we searched for candidate genes in this locus. As previously occurred in other parts of the POF critical region [14], [30] no genes with clear ovarian expression could be identified on the X chromosome, 500Kb to 1Mb from the breakpoint. However also in the 18q23 breakpoint region no genes highly expressed in the ovarian follicle could be found (not shown). It is therefore not unlikely that the 18q23 HCNEs could have a role also on the POF phenotype through ectopic expression of some of the genes involved in the breakpoint in the ovary and particularly in the ovarian follicle. The changes in chromatin modifications observed at the *SALL3* and *ATP9B* genes in patients lymphoblastoid cells may due to lack of the HCNE region upstream and of its controlling elements and support the hypothesis that chromatin alterations may be associated to the involvement of the HCNEs in the rearrangement.

In conclusion, the analysis reported indicated that the phenotype of the patient 263/96 could be the consequence of the involvement of HCNEs in the chromosomal rearrangement. Due to the abundance of HCNEs in the human genome [15], [16], we suggest that it might represents the first example of a common mechanism of disease, associated to isolated chromosomal rearrangements. In all cases when no gene involvement can be demonstrated we suggest that HCNEs should be searched.

## Materials and Methods

### Case

The girl, 263/96, was first seen at 3.2 years of age because of short stature. Her clinical characteristics were previously reported [13]. Clinical examination revealed no dysmorphic features, but a phenotype suggestive of congenital GHD including doll-like appearance with frontal bossing, poor development of the nasal bridge and increased adipose tissue of the trunk. Height was <−3 SDS and endocrine investigations were compatible with IGHD. At the time of puberty, primary amenorrhea, hypergonadotropic hypogonadism and ovarian dysgenesis were also found. Chromosome analysis on cultured lymphocytes and skin fibroblasts revealed that the patient carried a de novo X/18 translocation (46, X, t (X, 18) (q22.3, q23),inv(9)(p11q13). MRI of the H-P region showed posterior pituitary ectopia, anterior pituitary hypoplasia and pituitary stalk agenesis.

Written informed consent was obtained from the patient. The study was approved by the Institutional Review Board of the Policlinico San Matteo and the part regarding POF also by the Ethical Committee of the San Raffaele Hospital.

Lymphoblastoid cell line of the patient and of normal controls were grown as described [31].

### FISH mapping of the breakpoints

The 18q and Xq breakpoints in the patient 263/96 were mapped by FISH as described [32] using PAC and BAC clones from the Ensembl contig map (http://www.ensembl.org/). Fine mapping was done by Pulsed Field Gel Electrophoresis (PFGE) using restriction enzymes producing 50–100 Kb restriction fragments in genomic DNA [31]. Fragments were identified by Southern Blotting and hybridization to radioactively labeled probes prepared by PCR amplification of repeat free regions of genomic DNA (chr.X: 263×1F 5-GACACACTCTTAACACTGAGC and 263×1R 5-AGCTGATGGCTAGGAGAACC; chr.18: 263/5F 5-CATGCACGATACATCTGACC and 263/5R 5-AGAGGAGACAGAACATCTGG)

### Whole genome array-CGH

Whole genome array-CGH was performed by using the Agilent 244 K chip (Agilent Technologies, Palo Alto, CA, USA) according to the manufacturer's protocol. This genome-wide chip has an average resolution of about 20 Kb. Reference and patient DNAs were double-digested with *RsaI* and *AluI* for two hours at 37°C and labeled with the with Cy3-dUTP and Cy5-dUTP respectively. After column purification, the two differentially labeled DNAs were combined, denaturated and pre-annealed with 50 µg of Cot-1 DNA. Hybridization was performed at 65°C with rotation for 40 hours. After two washing steps, images of the arrays were acquired with the Agilent scanner and analyzed by using the Feature Extraction software (v9.1). A graphical overview of the results was obtained with CGH Analytics software (v3.4).

### Chromatin preparations and immunoprecipitation

Tissues were cut in small pieces in cold PBS 1× and fixed in 1% formaldehyde for 20 min at room temperature (RT). Lymphoblastoid cell lines were fixed in RPMI containing 1% formaldehyde for 20 min at RT. The reaction was quenched with 125 mM glycine and the cells pelletted. Brain pieces were homogenized with a douncer, centrifugated at 1200 rpm 5′ at RT, resuspended in cell lysis buffer (100 mM TrisHCl, 10 mM NaCl, 0.2% NP40) for 10′ in ice, centrifugated at 5000 rpm 5′ at RT, resuspended in Lysis Buffer (1%SDS, 10 mM EDTA pH8, 50 mM Tris-Hcl pH 8.1, 1 mM PMSF (Phenylmethylsulphonyl fluoride)) and sonicated with a XL2020 sonicator microtip (Misonix Incorporated) 20% power for 150s, to break DNA in chromatin to a size between 500 and 2000 bp. Chromatin was diluted 10 times in DB buffer (1% Triton, 2 mM EDTA pH 8, 150 mM NaCl, 20 mM Tris-Hcl pH 8.1, 1 mM PMSF). 100/200 µgr of chromatin was immunoprecipitated with 2 µl of antibody specific for acetylated histone H3 (Upstate Biotechnology, Billerica, MA, USA (UB 06-559)), 5 µl acetylated Histone H4 (UB 06-866), 5 µl di-methylated Lys4 Histone H3 (UB 07-030) and 5 µl tri-methylated Lys27 Histone H3 (UB 07-449), ON at 4°C and then mixed with 20 µl protein A sepharose beads (GE Healthcare, UK) saturated ON at 4°C with 500 µg of salmon sperm DNA and 100 µg of BSA and incubated for 3h at 4°C with gentle rocking. In parallel, immunoprecipitations with an unrelated antibody (anti urokinase plasminogen activator receptor) or no antibodies (Mock) were also performed as control. Beads were washed 5 times with Wash buffer (0.1% SDS, 1% Triton, 150 mM NaCl, 20 mM Tris-HCl pH 8.1, 1 mM PMSF) and 3 times with Final wash (0.1% SDS, 1% Triton, 2 mM EDTA, 500 mM NaCl, 20 mM Tris-HCl pH 8.1, 1 mM PMSF). Bound chromatin was eluted with 1% SDS and 0.5 µgr/µl Proteinase K for 3 hours at 50°C. DNA was de-crosslinked ON at 65°C and purified by phenol/chloroform extraction.

### Real time PCR and data analysis

DNA was analyzed by Real time PCR with Syber Green Universal Mix (Sigma-Aldrich, St.Louis, MO, USA) on a Light Cycler 480 (Roche Diagnostic, Basel, Switzerland). Total DNA, IP DNA and Mock IP DNA were quantified using picogreen (Molecular Probes, Carlsbad, CA, USA) and equal amounts of DNA (0.5 ng) were used in PCR. Samples were run in duplicate and each experiment was repeated two to five times, on independent preparations. Data quantification was done as described by Litt [33]. In brief, we determined the quantity of the template at the Ct (target cycle) and the efficiency (X) by a internal standard control and the fold increase was calculated as X^(inputCt-IPCt)^. All the values were corrected for nonspecific signal, by subtracting the values of Mock IP. All the PCR primers are listed in Table S2 in supplementary information.

### Transient transfection and luciferase reporter assay

HCNE were amplified from human DNA with PFU DNA polymerase (Stratagene, La Jolla, CA, USA) and cloned into pGL2 promoter vector (Promega, Madison, WI, USA) utilizing MluI and XhoI primer adaptor sequences (Table S1 in supplementary information). All clones were sequence verified.

DNA transfections were carried out using Lipofectamine 2000 following manufacturer's instruction (Invitrogen, Carlsbad, CA, USA). For luciferase reporter assay, cells were seeded in 96-well plates 24 h before transfection in DMEM, 10% fetal bovine serum. They were transfected at 90% confluence with 250 ng of plasmid/well, and 5 ng of renilla vector (Promega, Madison, WI, USA). 24 hours after transfection, the cells were harvested and the luciferase activity was determined using Dual-Glo luciferase assay system according to manufacturer's instructions (Promega, Madison, WI, USA). Luciferase activities were normalized to the renilla vector activity. Statistical analysis was done utilizing two tailed Student *t* test.

### RT-PCR and Real Time PCR

Total RNA was prepared from tissues using the RNA extraction kit Rnaeasy (Qiagen, Germany). One µg of total RNA was reverse transcribed in a 20 µl reaction using M-MLV reverse transcriptase (Invitrogen, Carlsbad, CA, USA). Primers for amplification of *Atp9b*, *Sall3* and *Chrdl1* are listed in Table S3, in supplementary information. Quantitative Real Time PCR (qRT-PCR) was performed with SYBR Green PCR Master Mix (Sigma-Aldrich, St.Louis, MO, USA) in a Light Cycler 480 (Roche Diagnostic, Basel, Switzerland). Samples were run in triplicates and the efficiency of each primer was calculated utilizing an internal standard control. All values were normalized for histone H3.

### In situ hybridization

In situ hybridizations were done as described [34].

## Supporting Information

Table S1 (0.01 MB XLS)

Table S2 (0.01 MB XLS)

Table S3 (0.01 MB XLS)
